# Meiosis, Balbiani body and early asymmetry of *Thermobia* oocyte

**DOI:** 10.1007/s00709-016-0978-7

**Published:** 2016-05-14

**Authors:** Waclaw Tworzydlo, Magdalena Marek, Elzbieta Kisiel, Szczepan M. Bilinski

**Affiliations:** Department of Developmental Biology and Morphology of Invertebrates, Institute of Zoology, Jagiellonian University, Krakow, Poland

**Keywords:** Oocyte, Meiosis, Prophase I, Bouquet stage, Lampbrush chromosomes, Nucleolar organizer

## Abstract

The meiotic division guarantees maintenance of a genetic diversity; it consists of several stages, with prophase I being the longest and the most complex. We decided to follow the course of initial stages of meiotic division in ovaries of *Thermobia domestica* using modified techniques of squash preparations, semithin sections, and electron microscopy. We show that germaria contain numerous germline cells that can be classified into three categories: cystoblasts, meiotic oocytes, and growing previtellogenic oocytes. The cystoblasts are located most apically. The meiotic oocytes occupy the middle part of the germarium, and the previtellogenic oocytes can be found in the most basal part, near the vitellarium. Analyses of the semithin sections and squash preparations show that post leptotene meiotic chromosomes gather in one region of the nucleoplasm where they form the so-called bouquet. The telomeres of the bouquet chromosomes are attached to a relatively small area (segment) of the nuclear envelope. Next to this envelope segment, the nucleolar organizers are also located. We show that in concert to sequential changes inside the oocyte nuclei, rearrangement of organelles within the ooplasm (oocyte cytoplasm) takes place. This leads to the formation of the Balbiani body and consequent asymmetry of the ooplasm. These early nuclear and cytoplasmic asymmetries, however, are transient. During diplotene, the chromosome bouquet disappears, while the Balbiani body gradually disperses throughout the ooplasm. Finally, our observations indicate the presence of lampbrush chromosomes in the nuclei of previtellogenic oocytes. In the close vicinity to lampbrush chromosomes, characteristic spherical nuclear bodies are present.

## Introduction

Meiosis reduces number of chromosomes by half. This process occurs in all sexually reproducing eukaryotes (Bernstein and Bernstein 2010; Bernstein et al. 2011). In meiosis, DNA replication is followed by two rounds of cell division resulting in the formation of four daughter cells, each with a halved number of chromosomes in comparison to the original parent cell. Two subsequent rounds of divisions are called meiosis I and meiosis II, respectively. Meiosis I and II are each divided into four consecutive stages: prophase, metaphase, anaphase, and telophase. Before the meiotic division, during the S phase of the cell cycle, the DNA is replicated, and therefore the chromosomes during meiosis consist of two identical sister chromatids, which remain joined together. After the DNA replication, meiotic cells enter G2 phase which can be prolonged, and in fact, it is the preparation for the division (Pawlowski and Cande 2005; Dumont and Brunet 2010). Because the number of chromosomes is halved during meiosis, gametes can fuse during fertilization to form a diploid zygote containing two copies of each chromosome, one from each parent. During the oogenesis, i.e., formation of the female gamete (egg cell), meiosis is arrested twice. The first arrest occurs at the end of prophase I. This phase is the longest and most complex of all meiotic phases and consists of following stages: leptotene, zygotene, pachytene, diplotene, and diakinase (diktiotene). The second arrest occurs later during metaphase II.

The ovaries of insects are composed of elongated units, termed the ovarioles (see Buning 1994; Bilinski 1998 for further details). An individual ovariole, as a rule, comprises three easily recognizable zones (regions): a terminal filament, germarium, and a vitellarium. The terminal filament is a simple stack of somatic cells, and it joins the ovariole (and the whole ovary) to the fat body lobes or to the body wall. The germarium contains dividing and differentiating germline cells, and the vitellarium consists of several developing ovarian follicles in a linear arrangement. Two basic categories of insect ovarioles are traditionally distinguished, the panoistic and meroistic (Buning 1994). In the panoistic ovarioles, all the germline cells develop into the oocytes (egg cells). In the meroistic ones, some of the germline cells differentiate into oocytes, while others become highly specialized nurse cells. These cells synthesize and subsequently transport macromolecules and organelles to the growing oocyte (for a review see Buning 1994; Matova and Cooley 2001).

The female reproductive system of *Thermobia domestica* is composed of two ovaries attached to paired lateral oviducts that join together to form a single common oviduct (Tworzydlo et al. 2014). Each of the ovaries is composed of five ovarioles of the panoistic type. Our previous studies have shown that in *Thermobia* the germaria contain three categories of germline cells, namely the cystoblasts (progenitor cells of the oocytes), early meiotic and previtellogenic oocytes, and that the chromosomes of post leptotene oocytes gather in one region of the nucleoplasm where they form the so-called bouquet (Tworzydlo et al. 2014; see Harper et al. 2004; Tomita and Cooper 2006; Chikashige et al. 2006; Ding et al. 2007 for further reading). We have shown additionally that in concert to the formation of the chromosome bouquet, rearrangement of organelles within the ooplasm (oocyte cytoplasm) takes place, leading to the formation of a complex organelle assemblage, known as the Balbiani body (Tworzydlo et al. 2014; for a review of the Balbiani body morphology and functioning see Kloc et al. 2004a, 2014; Pepling et al. 2007; Marlow and Mullins 2008). Interestingly, the Balbiani body locates invariably next to this segment of the nuclear envelope to which the telomeres of the bouquet chromosomes are attached (Tworzydlo et al. 2014). In this context, we decided to analyze sequential changes within the nucleoplasm during early stages of meiotic division in this insect. We show that transient asymmetry of the oocyte nucleus involves not only polar attachment of bouquet chromosomes, but also asymmetrical (eccentric) localization of nucleolar organizers.

## Material and methods

### Animals

Cultures of *Thermobia domestica* were maintained at 37 °C and 60 % relative humidity (RH) in plastic boxes containing test tubes filled with water and mixed oat flakes, powder milk, and dried water fleas (see Kisiel and Klag 2001 for further details).

### Light and electron microscopy

The ovaries were dissected under a Nikon SMZ1500 stereoscopic microscope (Nikon, Japan). They were fixed in a mixture of 2 % formaldehyde and 2.5 % glutaraldehyde in 0.1 M phosphate buffer, pH 7.3 for several days. Isolated ovarioles were rinsed and postfixed in 2 % osmium tetroxide and 0.8 % potassium ferrocyanide in the same buffer for 30 min at 4 °C. After dehydration in the series of ethanol and acetone, the material was embedded in Glycid Ether 100 (Epon 812) resin (Serva, Heidelberg, Germany). Semithin sections (0.7 μm thick) were stained with 1 % methylene blue and examined under a Leica DMR (Heidelberg, Germany) or Nikon Eclipse Ni (Nikon, Japan) microscopes. Ultrathin sections (80 nm thick) were contrasted with uranyl acetate and lead citrate according to standard protocols and analyzed with a Jeol JEM 2100 transmission electron microscope at 80 kV.

### AgNOR technique (silver impregnation)

The ovaries were dissected and fixed in a modified fixative solution which contained a mixture of 3 % formaldehyde and 1.5 % glutaraldehyde in 0.1 M phosphate buffer, pH 7.3 for 30 min. They were rinsed with PBS, dehydrated in series of ethanol and embedded in a histocryl acrylic resin (Agar Scientific Ltd, Stansted, Essex, UK). The staining of semithin histocryl sections was performed according to Howell and Black (1980) modified by Bilinski and Bilinska (1996). The sections were stained for 17 min at 37 °C with a 1:2 mixture of 2 % gelatin in 1 % formic acid and 50 % AgNO_3_ (Sigma, St. Louis, MO, USA). After rinsing with distilled water, the slides were analyzed under a Leica DMR or Nikon Eclipse Ni microscopes.

### DNA localization

Semithin histocryl sections (see above) were stained with Hoechst 33342 (1 μg/ml; Molecular Probes, Eugene, OR, USA) in the darkness, for 40 min, washed in distilled water and analyzed with a Leica DMR fluorescence microscope (FM), equipped with appropriate filters.

### Squash preparations

Gonads were dissected in several drops of PBS and immediately fixed in a Carnoy solution (3:1 ethanol-acetic acid mixture) for an hour at −20 °C. The ovarioles were separated and chromosomal preparations were made by squashing them in 45 % acetic acid; after freezing, the coverslips were taken off with a razor blade and preparations were air-dried. For morphology of chromosomes, the preparations were stained with 4 % Giemsa solution in phosphate buffer (pH 6.8) and analyzed with a Leica DMR or Nikon Eclipse Ni light microscopes. On several preparations, silver impregnation (AgNOR technique, see above) was performed. Briefly, the preparations were stained for 25 min at 37 °C with a 1:2 mixture of 2 % gelatin in 1 % formic acid and 50 % AgNO_3_ (Sigma, St. Louis, MO, USA). After rinsing with distilled water, the slides were analyzed under the light microscopes. Finally, some preparations were stained with Hoechst 33342 (see above) in the darkness, for 20 min, and analyzed with a Leica DMR fluorescence microscope (FM) equipped with appropriate filters.

## Results

### Morphology

**Fig. 1:**
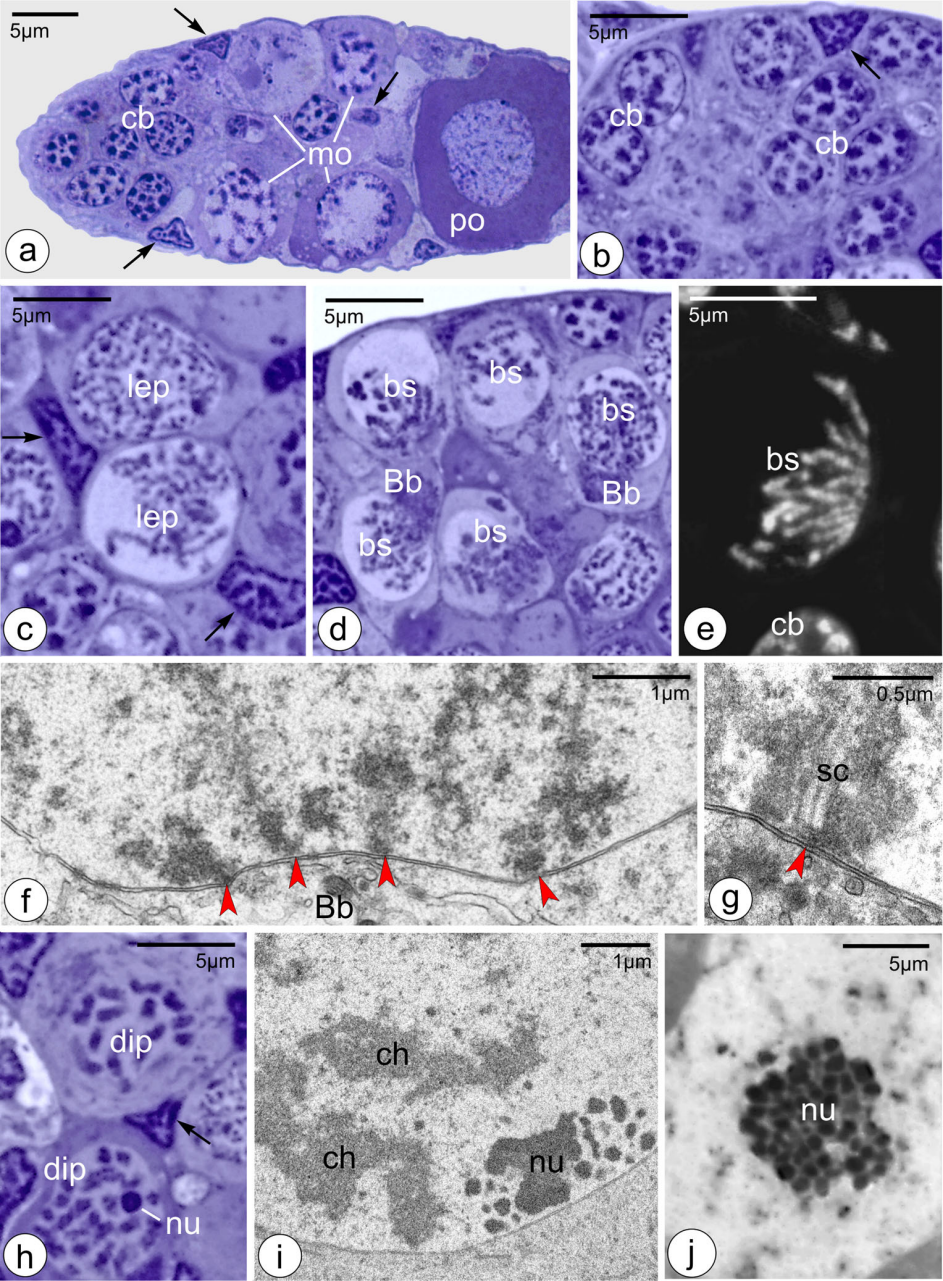
Morphology of germline cell present in the germarium. **a** Gross morphology of the germarium. Note numerous cystoblasts (*cb*) located most apically and magnified in **b**, meiotic oocytes (*mo*) that occupy the middle part of the germarium and previtellogenic oocyte (*po*) in the most basal part. **c**–**e**, **h** Meiotic oocytes in various stages of prophase I. *Bb* Balbiani body, *bs* bouquet stage, *cb* cystoblasts, *dip* diplotene, *lep* leptotene, *nu* nucleolus, and *arrows* indicate somatic cells. **f** Electron micrograph of an early bouquet stage oocyte. Note chromosome telomeres attached to nuclear envelope (*arrowheads*). **g** Electron micrograph of a late bouquet stage oocyte. Note synaptonemal complex (*sc*) in contact with the telomere and its attachment site to the nuclear envelope (*arrowhead*). **i**, **j** Nucleus of early diplotene oocyte **i** and previtellogenic oocyte **j**. Note that nucleoli (*nu*) consist of several smaller subunits. *ch* chromosomes. **a**–**d** and **h**, **j** Semithin sections stained with methylene blue; **e** semithin section stained with Hoechst 33342; **f**, **g** and **i** TEM

**Fig. 2:**
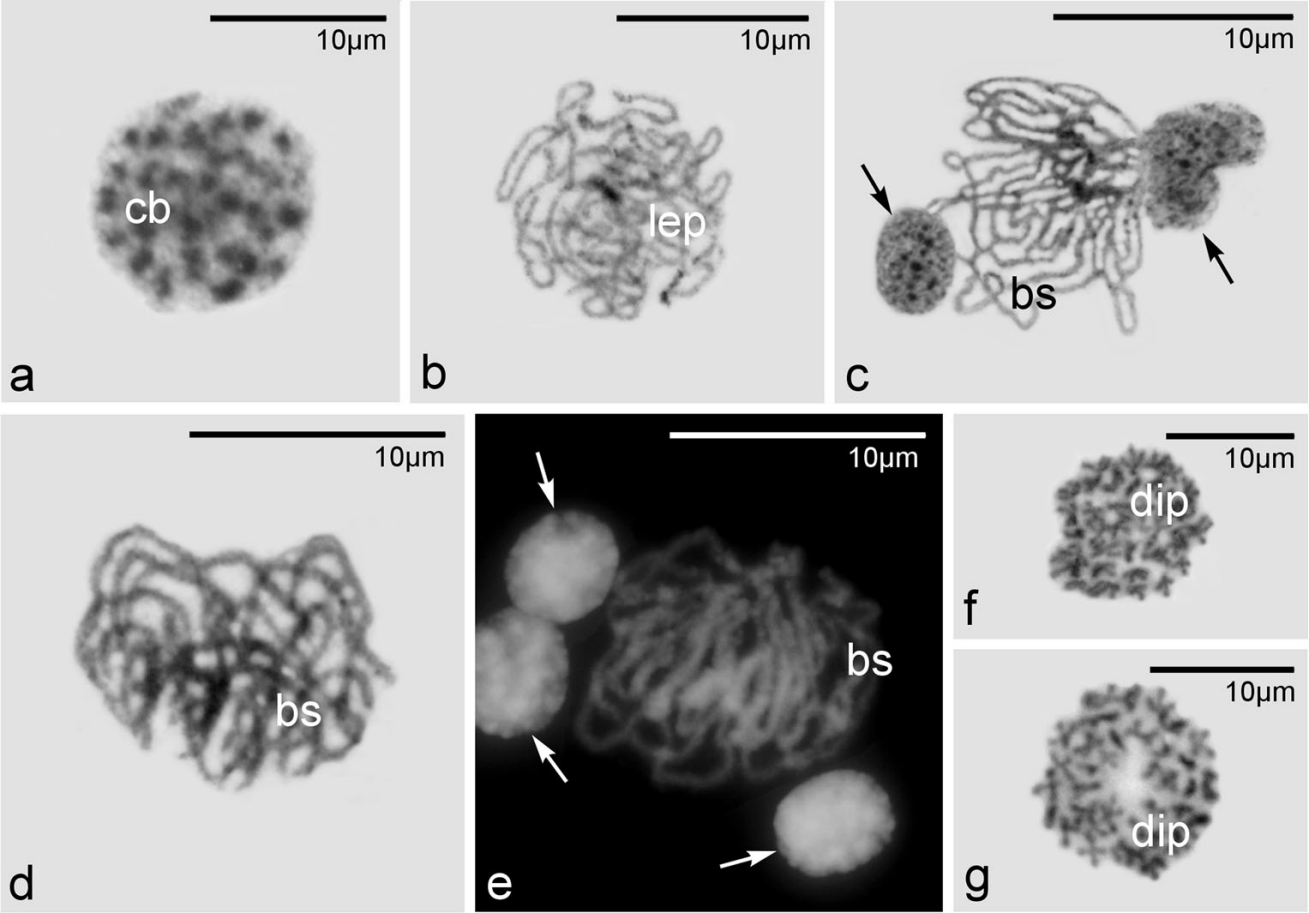
Subsequent stages of meiotic prophase I. Visualization of chromosomes in meiotic oocytes in various stages of prophase I and their progenitor cell, the cystoblast (*cb*), *bs* bouquet stage, *dip* diplotene, *lep* leptotene, and *arrows* indicate somatic cells. **a**–**d** and **f**, **g** Squash preparations stained with Giemsa; **e** squash preparation stained with Hoechst 33342

The germaria of *Thermobia* are relatively short and contain germline cells in various developmental stages, i.e., cystoblasts, early meiotic (leptotene–diploten) oocytes, and previtellogenic (diktiotene) oocytes, as well as small somatic cells (Tworzydlo et al. 2014). The chromatin of somatic cells is highly condensed and stains with the methylene blue (Epon sections), Giemsa, and Hoechst (squash preparations) (Figs. 1 and 2; arrows). The cystoblasts are located in the apical region of the germarium (Fig. 1a, b). Their nuclei are roughly spherical and contain prominent methylene blue and Giemsa-positive chromatin aggregations immersed in a relatively transparent karyoplasm (Figs. 1b and 2a). Central region of the germarium contains early (prophase I) meiotic oocytes. During early leptotene, the chromosomes are genuinely thin (up to 0.03 μm width); they are distributed more or less evenly throughout the nucleoplasm (Figs. 1c and 2b). At the leptotene-zygotene transition, all the oocyte chromosomes gather in one region of the nucleoplasm where they form the bouquet. This stage will be referred to as the early bouquet. The chromosomes of the early bouquet are slightly thicker (0.05–0.07 μm in width). Analysis of histocryl sections stained with Hoechst and squash preparations showed that the bouquet chromosomes form characteristic loops and that their telomeres are clustered and attached to a relatively small area of the inner membrane of the nuclear envelope (Figs. 1d–f and 2c–e). During late zygotene, the synaptonemal complexes are formed between coupled chromosomes (Fig. 1g). We will refer to this stage as the late bouquet. Figure 1g shows the synaptonemal complex in contact with the telomere and its attachment site to the nuclear envelope. Simultaneously, to the gradual formation of the chromosome bouquet in the nucleoplasm, rearrangement of organelles within the ooplasm (oocyte cytoplasm) takes place. This process leads to the formation of the complex assemblage of organelles known as the Balbiani body (Tworzydlo et al. 2014). During the next stage, i.e., the diplotene, the bouquet disappears, the chromosomes become progressively shorter and markedly thicker (0.6–0.7 μm) (Figs. 1h and 2f, g). Concurrently, the nucleolus is formed in the nucleoplasm (Fig. 1h, i). Careful analysis of serial ultrathin sections revealed that the nucleolus arises next to the nuclear envelope (Fig. 1i). As nucleologenesis progresses, the nucleolus drifts off from the envelope and locates more centrally (Fig. 1h).

The most basal part of the germarium contains large previtellogenic (diktioten) oocytes. Their nuclei are surrounded by slightly irregular nuclear envelope and contain filiform aggregations of chromatin (Fig. 1j), prominent nucleoli, and spherical nuclear bodies (see Dundr and Misteli 2010; Mao et al. 2011 for the discussion of the biogenesis and functioning of the nuclear bodies) immersed in a transparent nucleoplasm (Fig. 1a, j). The nucleoli consist of several irregular subunits (Fig. 1j). Such an organization of the nucleoli has been observed in panoistic ovaries of several hemimetabolous insects (e.g., Pritsch and Buning 1989; Rosciszewska and Soldan 1999). It is usually believed that it reflects high transcriptional activity of ribosomal genes (rDNA). During early previtellogenesis, the Balbiani body is no longer recognizable, and its constituents disperse throughout the ooplasm.

### Analysis of semithin histocryl sections and squash preparations stained with AgNOR method

**Fig. 3:**
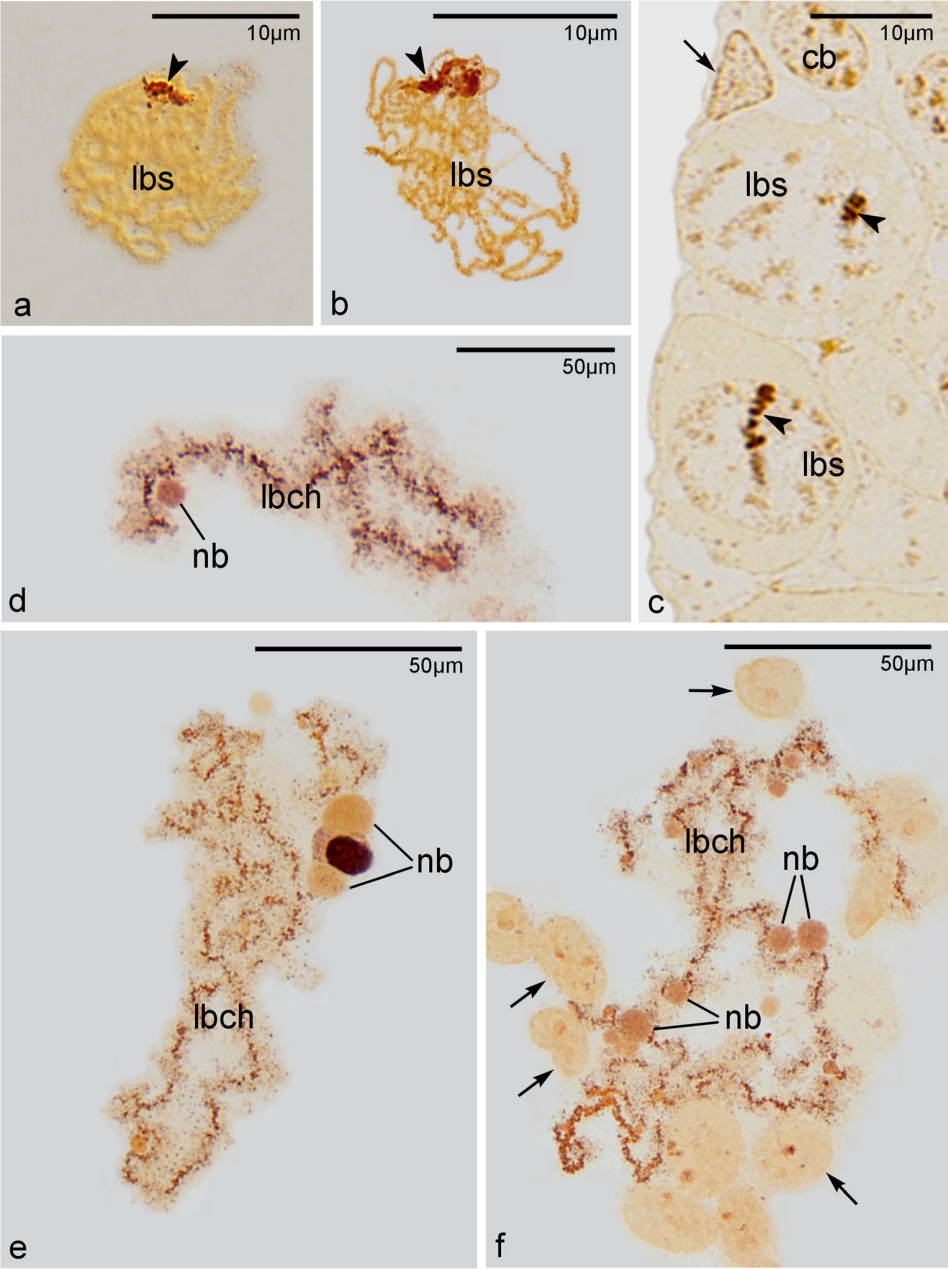
Distribution of AgNOR proteins. Squash preparations **a**–**b** and **d**–**f** and semithin section **c** stained with AgNOR technique. **a**–**b** Meiotic oocytes in late bouquet stage (*lbs*). **c** Middle part of the germarium. Note cystoblasts (*cb*) and late bouquet stage oocytes (*lbs*). *Arrowheads* point to asymmetrically localized nucleolar organizers; *arrow* indicates somatic cell. **d**–**f** Lampbrush chromosomes (*lbch*) accompanied by spherical nucleolar bodies (*nb*). *Arrows* indicate somatic cells

Our histological analyses (see above) indicated that the oocyte nucleoli reappear during early diplotene stage and that they are always formed next to the nuclear envelope. In this context, we decided to analyze the distribution of AgNOR proteins (proteins present in the nucleolar organizer region; see Pikaard 2000; Andersen et al. 2005 for further reading) on squash preparations of early meiotic oocytes. Interestingly, this technique clearly showed that during late bouquet stage the nucleolar organizers are located asymmetrically—invariably in the vicinity of the clustered telomeres (Fig. 3a, b; arrowheads). Staining of semithin histocryl sections with the AgNOR method confirmed this observation and indicated that the nucleolar organizers are attached (as telomeres are) to the nuclear envelope (Fig. 3c; arrowheads). Thus, the asymmetry of early meiotic oocytes of *Thermobia* is defined not only by the position of the telomeres of the bouquet chromosomes but also by the position of the nucleolar organizers.

Analysis of squash preparations stained with AgNOR method indicated additionally that chromosomes of diktiotene (previtellogenic) oocytes are in lampbrush state (Fig. 3d–f). This staining technique showed typical constituents of lampbrush chromosomes: strongly AgNOR positive axial elements and slightly positive, laterally extended loops (Fig. 3d–f). The axial elements are about 1.5 μm thick, while the diameter of whole chromosomes (with lateral loops) usually exceeds 6 μm. In the neighborhood of lampbrush chromosomes or in contact with them, characteristic spherical bodies were often present (Fig. 3d–f). As a rule, these bodies were only slightly stained; however, some of them exhibited strong AgNOR positive reaction (Fig. 3e). The nature of the former ones remains unclear, whereas the latter most probably represent transcriptionally active nucleoli.

## Discussion

In the vast majority of vertebrates and invertebrates, initial stages of oogenesis involve formation of characteristic germline cysts (see King 1970; de Cuevas et al. 1997; Pepling and Spradling 1998, Pepling et al. 1999; Kloc et al. 2004b for further review). These cysts arise as a result of consecutive incomplete mitotic divisions of the progenitor cell, the cystoblast (Pepling et al. 1999; Ong and Tan 2010). Our previous studies showed that in *Thermobia*, the cystoblasts do not divide mitotically, and therefore, the syncytial germline cysts are never formed (Tworzydlo et al. 2014). Instead, the cystoblasts directly enter the meiotic prophase and start to differentiate into the oocytes (Tworzydlo et al. 2014).

Here, we show that in *Thermobia*, at the leptotene-zygotene transition meiotic chromosomes gather in one region of the nucleoplasm, where they form the so-called bouquet (Harper et al. 2004; Tomita and Cooper 2006; Chikashige et al. 2006; Ding et al. 2007). All the telomeres of bouquet chromosomes are clustered and attached to a small segment of the nuclear envelope. Somewhat later (during late bouquet stage), the synaptonemal complexes arise between homologous chromosomes. We show also that during late bouquet stage the nucleolar organizers locate next to the telomeres of the bouquet chromosomes. As a result, the nucleoplasm of bouquet stage oocytes becomes asymmetrical (polar). Simultaneously, on the cytoplasmic side of this segment of the nuclear envelope to which the telomeres and the nucleolar organizers are attached, the Balbiani body is formed. This organelle assemblage comprises mitochondria (forming a hyperfused mitochondrial network), Golgi cisternae, endoplasmic reticulum elements, and accumulation of nuage material (Kloc et al. 2014; Tworzydlo et al. 2014). In the light of these data, we suggest that the early asymmetrization of *Thermobia* oocytes involves two processes: formation of the chromosome bouquet and localization of the nucleolar organizers within the nucleoplasm and formation of the Balbiani body in the cytoplasm. It is obvious that these two processes (one occurring in the nucleoplasm, the other in the ooplasm) are related, and consequently, one of them must play a decisive role. In the preceding paper, we showed that the Balbiani body starts to form slightly earlier (in the cystoblasts) than the chromosome bouquet arises (Tworzydlo et al. 2016). If so, it might be speculated that the oocyte asymmetry is initially dictated by the position of the early Balbiani body, and that the mitochondria of this organelle assemblage deliver energy (ATP) for the movement of chromosome telomeres along the nuclear envelope. The latter idea agrees well with our previous experiments showing that membrane potential of the Balbiani body mitochondrial network is substantially higher than that of individual mitochondria located outside of the Balbiani body (Tworzydlo et al. 2016).

Our analyses show also that both nuclear and cytoplasmic polarities of *Thermobia* oocytes are transient and last only to the beginning of the previtellogenic growth (the diktiotene). At this phase, the Balbiani body disperses and gradually covers the entire perimeter of the oocyte nucleus (Kloc et al. 2014; Tworzydlo et al. 2014). The asymmetry of the nucleoplasm becomes indiscernible even earlier—at the onset of the diplotene when the bouquet chromosomes drift apart from each other and the nucleolus moves to the nucleus center (this study).

At the moment, we do not know whether transient polarity of *Thermobia* oocyte plays an instructive role during oogenesis and/or embryonic development; it is worth noticing, however, that similar transient association of the Balbiani body and bouquet chromosomes was described also in two model species, African clawed frog, *Xenopus laevis* (Kloc et al. 2004b) and zebrafish, *Danio rerio* (Elkouby et al. 2016).
